# Interactions of the chemokines CXCL11 and CXCL12 in human tumor cells

**DOI:** 10.1186/s12885-022-10451-4

**Published:** 2022-12-20

**Authors:** Christian Koch, Nina Charlotte Fischer, Malte Puchert, Jürgen Engele

**Affiliations:** 1 Institute of Anatomy, University of Leipzig, Medical Faculty, Liebigstr. 13, 04103 Leipzig, Germany; 2 Department of Medical Oncology and Hematology, University of Zurich and University Hospital of Zurich, Raemistrasse 100, 8091 Zurich, Switzerland

**Keywords:** Chemokine receptors, CXCR4, CXCR7, CXCR3, CXCL11, CXCL12, Combined effects, Tumor progression, Tumor metastasis, Tumor growth

## Abstract

**Background:**

The chemokines, CXCL12 and CXCL11, are upregulated in tumors from many organs and control their progression. CXCL12 and CXCL11 affect tumor cell functions by either binding their prime receptors, CXCR4 and CXCR3, respectively, and/or CXCR7 as a common second chemokine receptor. In humans, CXCR3 exists in the functional splice variants, CXCR3A and CXCR3B, which either have pro- or anti-tumor activity, respectively. Despite the intimate crosstalk between the CXCL12- and CXCL11-system, the impact of a combination of CXCL12 and CXCL11 on tumor progression remains vague.

**Methods:**

In the present work, we have analyzed CXCL12 and CXCL11 for combined effects on migration, invasion, proliferation, and cytostatic-induced apoptosis of the human tumor cells, A549, A767, A772, DLD-1, and MDA-MB-231.

**Results:**

We demonstrate that the mode of interaction differs with respect to cell type and function and allows for either potentiation, attenuation or no changes of cellular responses. The divergent responses are not the result of the distinct use of different CXCL12- and CXCL11-receptors by the respective tumor cells, but in case of cell migration seem to be associated with the activation of p38 signaling pathways.

**Conclusions:**

Our findings point to therapeutic limitations of ongoing efforts to selectively target CXCR3, CXCR4, or CXCR7 in cancer patients, and rather favor individualized targeting strategies.

**Supplementary Information:**

The online version contains supplementary material available at 10.1186/s12885-022-10451-4.

## Background

The CXCL12 chemokine system regulates tumor formation and progression in numerous organs [1]. In some of these tumors, expression levels of CXCL12 further emerged as valuable prognostic biomarkers [2–4]. Specifically, CXCL12 controls tumor progression by binding to its receptors, CXCR4 and/or CXCR7, and subsequently affecting tumor cell survival, proliferation, and migration. These effects are predominantly achieved via activation of ERK-, AKT- and/or p38 MAPK-signaling [1, 5]. Another chemokine involved in the control of tumor progression is CXCL11 [6]. CXCL11 binds CXCR3, but also CXCR7, and, hence, closely interweaves the CXCL11-system with the CXCL12-system [7]. Additional ligands for CXCR3 are CXCL4, CXCL9, and CXCL10 which, however, bind the receptor protein with distinctly lower affinity than CXCL11 [8] and in addition activate different signaling pathways [9]. In humans, CXCR3 exists in the splice variants, CXCR3A, CXCR3B, and CXCR3alt, which have partly adverse effects on tumor progression. Whereas CXCR3A exerts pro-tumor activity and for example promotes tumor cell proliferation and migration, CXCR3B has anti-tumor activity and inhibits tumor cell proliferation and migration [10]. In addition to their direct effects on tumor cell function, CXCL12 and CXCL11 indirectly control tumor progression by modulating the tumor microenvironment, thereby affecting immune responses and tumor angiogenesis [11, 12].

Although, evidence exists that CXCL12 and CXCL11 exert combined effects during tumor progression, putative interactions are currently not well characterized. Indeed, in many organs, including breast, ovary, prostate, lung, kidney, as well as within the gastrointestinal tract, tumor progression is likewise affected by CXCR4, CXCR3, and CXCR7 [10, 13]. Moreover, several of these tumors show increased expression of both CXCL12 and CXCL11 [14]. Finally, our previous work showed that in many tumors, CXCR7 is essential for both CXCL12- and CXCL11-induced cell migration [6, 15].

We have now analyzed CXCL12 and CXCL11 for combined effects on tumor progression. In addition, we have asked whether the mode of interaction would be specified by the distinct (set of) CXCL12- and CXCL11-receptors employed by respective cells. We demonstrate that CXCL12 and CXCL11 interact to control migration, invasion, and survival of most, but not of all tumor cells. Depending on the cell type and respective cell function, this interaction allows for either enhanced or reduced cellular responses. These diverse effects do not seem to correlate with the use of distinct chemokine receptors by tumor cells.

## Methods

### Cell cultures

The human tumor cell lines, A549 (lung adenocarcinoma), DLD-1 (colorectal adenocarcinoma), and MDA-MB-231 (breast adenocarcinoma) were purchased from ATCC. The long-term human glioma cultures, A767 and A772 [16, 17], have been generously provided by Dr. Rolf Mentlein (Kiel, Germany). All cells were propagated in either DMEM (4.5 g/l glucose; Gibco, Life Technologies, Carlsbad, CA; A767, A772, MDA-MB-231) or RPMI 1640 (Gibco; A549, DLD-1), supplemented with 0.05% gentamycin (Gibco) and 10% fetal bovine serum (FBS; Gibco). Cells were plated on 10 cm culture dishes (TPP, Trasadingen, Switzerland). Subconfluent cultures were used for experiments. For immunofluorescent labeling, cells were seeded on glass coverslips (Thermo Fisher Scientific, Dreieich, Germany) previously coated with poly-L-ornithine (0.4 μg/ml; Sigma, St. Louis, MO) for 24 h.

### Western blot analysis

For Western blot analysis, cultured cells were lysed in 2% SDS and protein content was determined using the Pierce™ BCA Protein Assay Kit (Thermo Fisher) according to the manufacturer’s instructions. Proteins (10–20 μg/lane) were separated by SDS-(10%) polyacrylamide gel electrophoresis and transferred to nitrocellulose by electroblotting. If required, activated sodium orthovanadate (10%) was added to the lysis buffer to avoid protein dephosphorylation. After blocking non-specific binding sites with 5% bovine serum albumin or 5% skimmed milk for 60 min, blots were incubated overnight at 4 °C with the respective primary antibody (see additional file 1), following a 1-h-incubation with peroxidase-conjugated secondary antibodies (see additional file 1). Antibody-labeling was visualized with Pierce™ ECL Western Blotting Substrate (Thermo Fisher). To control for protein loading, membranes were reblotted with anti-GAPDH antibodies (see additional file 1). Chemiluminescence was captured on a Biostep Celvin S Imager (Biostep, Burkardtsdorf, Germany) and immunoreactive protein bands were quantified using the TotalLab 1D software (TotalLab, Newcastle-Upon-Tyne, UK).

### Chemotaxis assay

Migratory responses of cancer cells to CXCL11 and/or CXCL12 were analyzed using a modified 12-well Boyden chamber (Neuro Probe, Cabin John, MD) in which the upper and lower wells were separated by polyornithine coated Nucleopore® PVP-free polycarbonate filter (Whatman; Maidstone, UK; 8 μm pore size). Prior to analysis, cells were harvested and incubated for 1 h in serum-free medium, supplemented with one of the following chemokine receptor antagonists or (downstream) pathway inhibitors: AMG487 (CXCR3 antagonist; 10 μM; Tocris, Wiesbaden, Germany; dissolved in DMSO), CCX771 (CXCR7 antagonist; 100 nM; ChemoCentryx; Mountain View, CA; dissolved in DMSO), AMD3100 (CXCR4 antagonist; 10 μM; Sigma; dissolved in double-distilled water), PD98059 (MEK1-inhibitor; 20 μM; Cell Signaling Technology, Danvers, MA; dissolved in DMSO), LY294002 (PI3-kinase inhibitor; 20 μM; Cell Signaling Technology; dissolved in DMSO), SB203580 (p38 MAP kinase inhibitor; 10 μM; Cell Signaling Technology; dissolved in DMSO). For control purposes, untreated cultures were additionally supplemented with adequate concentrations of DMSO. For seeding, cells were counted using an improved Neubauer chamber and 10,000 cells were placed into the upper well of the Boyden chamber. The lower well received 150 μl of serum-free medium supplemented with CXCL11 (1 ng/ml to 100 ng/ml; Cell Guidance Systems, Cambridge, UK), and/or CXCL12 (1 ng/ml to 100 ng/ml; ALMAC, Craigavon, UK). In selected experiments, the lower well received a constant concentration (10 ng/ml) of either CXCL12 or CXCL11 and in addition varying concentrations (1 ng/ml to 100 ng/ml) of the respective other chemokine. As a positive control, 10% FBS was added to the lower wells. Chamber was incubated at 37 °C in a water- saturated atmosphere of 95% air and 5% CO_2_ for 4 h. After incubation, non-migrated cells, attached to the upper part of the membrane, were wiped off and migrated cells, attached to lower part of the membrane, were fixed with methanol, stained with DAPI (AAT Bioquest, Sunnyvale, CA), and counted on an Olympus BX40 microscope at 100x magnification using the Olympus cellSens Dimension software (Olympus, Shinjuku, Japan). Number of cells migrating in the absence of chemoattractants was set to 1. Migration index was calculated as the ratio of cells migrating in the presence and absence of chemokines.

### Invasion assay

To assess invasion behavior of tumor cells, Boyden chamber assay was performed with polycarbonate filters, previously coated with Matrigel (Corning, Acton, MA; 30 μl, further diluted 1:3 with culture medium) at 37 °C for 1 h. For seeding, cells were resuspended in either DMEM or RPMI containing 1% FBS and 0.05% gentamycin. Number of migrated cells was determined after 72 h as delineated above. Number of cells invading the membrane in the absence of chemokines was set to 1. Invasion index was calculated as the ratio of cells invading the membrane in the presence and absence of chemokines. To assess the role CXCR4, CXCR7, and CXCR3 in CXCL11- and/or CXCL12-dependent cell invasion, invasion assay was performed with cells preincubated with AMG487 (10 μM), CCX771 (100 nM), or AMD3100 (10 μM) for 1 h as described above.

### Apoptosis assay

Efficacy of different cytostatic agents to induce death of cancer cells was initially assessed by immunocytochemistry. For this purpose, cells were plated on glass cover slips and treated with different concentrations of doxorubicin (0.1 μM to 10 μM), cisplatin (10 μM to 40 μM) or temozolomide (10 μM to 200 μM; all obtained from the University of Leipzig Medical Center Pharmacy) for 3 h to 24 h. Cells were subsequently fixed and stained with DAPI and antibodies against cleaved-caspase-3 as delineated below. Apoptotic cells were counted on a Zeiss confocal laser scan microscope and expressed as per cent of total number of cells present in the same observation fields. To assess effects of chemokines on apoptotic cell death, cells were treated with 100 ng/ml of CXCL11 and/or CXCL12 24 h prior to the addition of cytostatics. After another 6 - 12 h, apoptotic cells were identified by the BD FITC Annexin V Apoptosis Detection Kit (Becton Dickinson, Franklin Lakes, NJ) and 10.000 events were quantified on a Becton Dickinson LSRFortessa™ flow cytometer. Data were analyzed using the FlowJo version 10 software (FlowJo; Ashland, Oregon). Apoptotic cell numbers present in cytostatic-treated cultures were set to 1.

### Gelatin zymography

For evaluation of gelatinase activity, cells were grown in serum-containing culture medium on 6-well plates with a change to serum-free medium 24 hours prior to treatment with either CXCL11 (100 ng/ml), CXCL12 (100 ng/ml), or both. Cell supernatant was collected after 48 h and concentrated with Amicon Ultra-2 Centrifugal filter units (Merck Millipore, Burlington, MA). Proteins were loaded (20–30 μg per lane) on polyacrylamide gels containing gelatin (1 mg/ml) and separated by electrophoresis under non-reducing conditions. Gels were subsequently incubated in activation buffer (2.5% Triton X-100, 50 mM Tris HCl, 5 mM CaCl_2_, 1 μM ZnCl_2_) for 24 h and stained with Coomassie-Blue for 1 h. Following destaining with methanol/acetic acid, gels were analyzed on a Biostep Celvin S Bioluminescence Detector. Recombinant activated matrix metalloproteinase (MMP)-2 (#550502, Biolegend, San Diego, CA) and recombinant activated MMP-9 (ab81550, Abcam, Cambridge, UK) were used as controls.

### Quantitative real-time-PCR (qRT-PCR)

Total RNA was extracted by TRI Reagent™ solution (Invitrogen, Carlsbad, CA), followed by reverse transcription of 1 μg RNA using Protoscript First Strand cDNA Synthesis Kit (Biolabs, Frankfurt, Germany) as specified by the manufacturer. For quantification of gene expression, qRT-PCR analysis was performed with Maxima SYBR**®** Green/ROX qPCR Master Mix (Thermo Fisher, Waltham, MA) on a CFX 96 Thermal Cycler system (Bio-Rad, Munich, Germany). Gene expression was calculated by the ΔΔCT method and normalized to β-actin. The following primers were used:

MMP-2, forward 5′- CTCAGATCCGTGGTGAGATCT-3′, reverse 5′- CTTTGGTTCTCCAGCTTCAGG-3′.

MMP-9, forward 5′-ATCCAGTTTGGTGTCGCGGAGC-3′, reverse 5′-GAAGGGGAAGACGCACAGCT-3′.

Beta-Actin, forward 5′-GGCCTCGCTGTCCACCTT-3′, reverse 5′-TGTCACCTTCACCGTTCCAGTTTT-3′.

### Immunocytochemistry

For immunostaining, cells were fixed with 4% paraformaldehyde in phosphate-buffered saline (PBS) for 15 min. To block unspecific binding sites and at the same time permeabilize cells, cultures were incubated with PBS containing 5% bovine serum albumin and 0.05% saponin for 1 h. Primary antibodies (see additional file 1) were applied overnight at 4 °C, followed by incubation with an appropriate Alexa Fluor 488- or Alexa Fluor 555-labelled secondary antibody (see additional file 1) at 37 °C for 1 h. Cultures in which the primary antibody was omitted served as controls. Following staining of cell nuclei with DAPI, cultures were mounted with Dako Glycergel Mounting Medium (Dako Inc., Carpinteria, CA) and analyzed on a Zeiss confocal laser scan microscope, using ZEN software (Carl Zeiss, Oberkochen, Germany).

### Proliferation assay

To assess effects of chemokines on cell proliferation, cells were seeded into 6-well culture plates and maintained with serum-free medium, supplemented with either CXCL11 (100 ng/ml) and/or CXCL12 (100 ng/ml). Medium, containing the respective chemokine(s) was renewed on day 2 and additionally supplemented with bromodeoxyuridine (10 μM; Becton Dickinson; Franklin Lakes, NJ). After a 2-h-incubation step, cells were harvested, fixed, and stained for BrdU using the BD FITC BrdU Flow Kit (Becton Dickinson, Franklin Lakes, NJ) according to the manufacturer’s instructions. Following labeling DNA with 7-AAD (BD, Franklin Lakes, NJ), cells were analyzed by flow cytometry using a Becton Dickinson LSR Fortessa.

### Statistics

Data, obtained from at least three experiments, are given as mean ± SD. One way analysis of variance (ANOVA) followed by pairwise multiple comparison procedures (Tukey post-hoc test) was used for statistical analysis (GraphPad). Differences with *p* < 0.05 were considered significant.

## Results

The present experiments were performed with the human tumor cell lines, A549, DLD-1, and MDA-MB- 231, which we previously characterized for expression as well as their use of CXCR4, CXCR3, and CXCR7 in CXCL12- and CXCL11-dependent chemotaxis [6, 15] (additional file 2). In addition, we included the human glioma cells, A767 and A772 into our analyses [16, 17]. These cells express CXCR7, CXCR4, and CXCR3 as demonstrated by Western blotting (additional file 3). Comparable to A549 cells, CXCL11- and CXCL12-induced migration of A772 cells depends on CXCR3/CXCR7 and CXCR7, respectively. Moreover, like with DLD-1 cells, CXCL11-dependent migration of A767 cells requires CXCR3, whereas unlike DLD-1 cells, CXCL12-induced migration of A767 cells depends on CXCR7/CXCR4 (additional files 2 and 4).

### Evaluation of combined effects of CXCL11 and CXCL12 on tumor cell migration

**Fig. 1:**
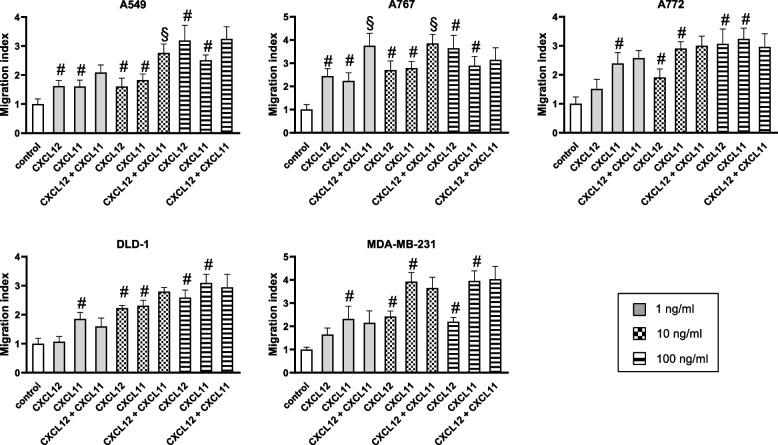
Combined effects of CXCL12 and CXCL11 on tumor cell migration. Chemotactic responses of tumor cells to the indicated concentrations of CXCL12, CXCL11, or a combination of both chemokines were assessed in a modified Boyden chamber for 4 h. The number of cells migrating in the absence of chemokines was set to 1. Migration index was calculated as the ration of cells migrating in the presence and absence of chemokines. Data represent average migration index (+SD) as determined in 4–9 experiments. CXCL12 and CXCL11 induced chemotaxis of the various tumor cells in a dose-dependent manner. At low to medium concentrations, CXCL12 in combination with CXCL11 additively promoted migration of A549 and A767 cells. # *p* < 0.05, single treatment vs. control. § *p* < 0.05 double treatment vs. single treatments

**Fig. 2:**
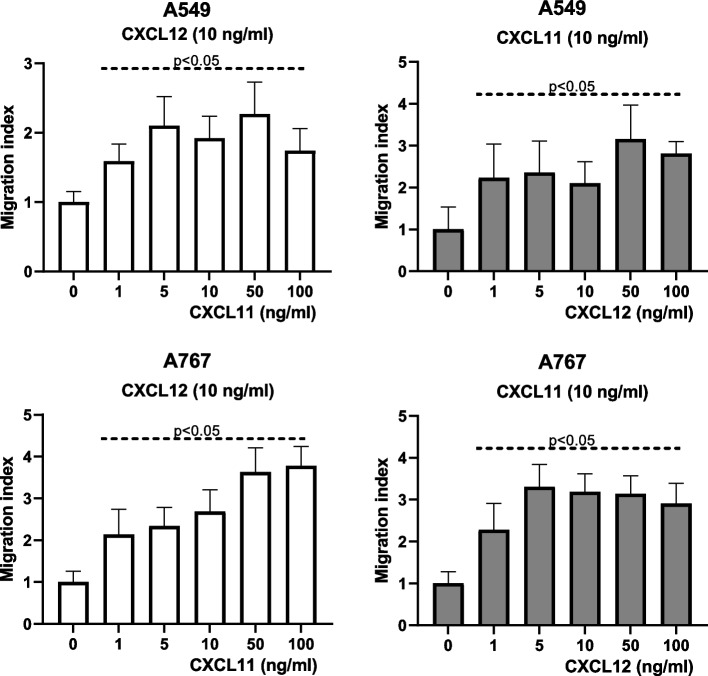
CXCL11 and CXCL12 synergistically promote migration of A549 and A767 cells. To define whether CXCL11 and CXCL12 additively or synergistically affect cell migration, chemotactic responses of A549 and A767 cells were determined in the presence of a constant concentration of one chemokine (10 ng/ml) and increasing concentrations of the respective other chemokine (1 ng/ml – 100 ng/ml). Migration index was assessed as described in Fig. 1. Data represent average migration index (+SD) as determined in 4–10 experiments. Addition of the respective other chemokine at concentrations as low as 1 ng/ml already increased migration by 1.6-fold to 2.3-fold, indicative for synergistic effects of chemokines on cell migration

To assess putative combined effects of CXCL11 and CXCL12 on cell migration, we assessed numbers of cells migrating in the presence of either CXCL12 or CXCL11 at concentrations of 1 to 100 ng/ml or a combination of both chemokines. CXCL12 and CXCL11 were almost equally potent in inducing migration of the various tumor cells, resulting in a dose-dependent, maximal 2.5–3.5-fold increase of the migration index (Fig. 1). Only MDA-MB-231 cells displayed a roughly 2-fold higher migratory response to maximal effective concentrations of CXCL11 when compared to maximal effective concentrations of CXCL12 (Fig. 1). FBS (10%), which served as a positive control, more potently increased migration of most tumor cells (A549, 8.0 + 0.9-fold; A767, 7.9 + 0.9-fold; A772, 7.4 + 1.5-fold; DLD-1, 4.4 + 0.5-fold; MDA-MB-231, 2.2 + 0.3; *n* = 5). All tumor cells showed statistically significant migratory responses to CXCL11 at concentrations as low as 1 ng/ml, whereas with CXCL12 at 1 ng/ml statistically significant migratory responses only occurred with A549 and A767 cells (Fig. 1). In contrast to single chemokines, CXCL12 and CXCL11 in combination more potently stimulated the migration of A549 and A767 cells, but not of the other tumor cells. Of note, in both tumor cell types enhanced migratory responses only occurred with low to medium concentrations (1–10 ng/ml), but not with high concentrations (100 ng/ml) of the chemokines. To determine whether the combined effects of CXCL12 and CXCL11 on the migration A549 and A767 cells are additive or synergistic in nature, we tested migratory cell responses to constant concentrations (10 ng/ml) of one chemokine and increasing concentrations (1 ng/ml to 100 ng/ml) of the other chemokine (Fig. 2). We defined additive effects as the sum of single effects and synergistic effects as a surplus of the sum. We found that addition of concentrations as low as 1 ng/ml of the respective other chemokine was sufficient to increase the migration index of A549 and A767 cells by 1.6-fold to 2.3-fold, implying that CXCL12 and CXCL11 exert synergistic effects on cell migration.

Together, these findings establish that CXCL12 and CXCL11 synergistically control migration of some, but not of all tumor cells. Since A549 and A767 cells use distinctly different receptor(s) combinations to mediate migratory responses of CXCL12 and CXCL11 (see additional files 2 and 4), these findings further imply that the occurrence of combined effects does not depend on the functional organization of the CXCL12- and CXCL11-systems in individual tumor cells.

### Signaling molecules/pathways involved in tumor cell migration

**Fig. 3:**
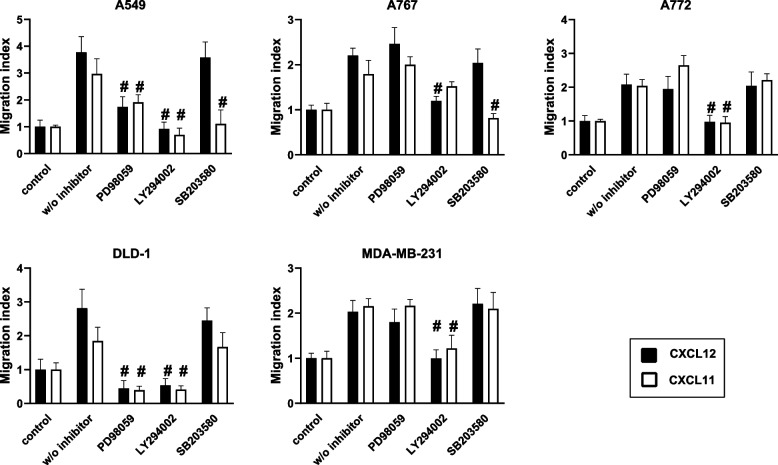
Signaling pathways mediating the chemotactic responses of tumor cells to CXCL12 and CXCL11. Cultured tumor cells were maintained in the presence or absence of either PD98059 (20 μM), LY294002 (20 μM), or SB203580 (10 μM) for 1 h, and subsequently tested for their migratory responses to CXCL12 (100 ng/ml) and CXCL11 (100 ng/ml) in a modified Boyden chamber as described in Fig. 1. Data represent average migration index (+SD) as determined in 4–10 experiments. Depending on the cell type, chemotactic responses to CXCL12 and CXCL11 differentially involve ERK, PKA, and/or p38. Within a given cell type CXCL12- and CXCL11-induced chemotaxis either depends on identical or different signaling molecules/pathways. #*p* < 0.05, presence vs. absence of inhibitor

Since synergistic effects of CXCL12 and CXCL11 on tumor cell migration do not correlate with the selective use of distinct chemokine receptors / receptor combinations, we asked whether additive effects would correlate with receptor-activated signaling pathways. Signaling molecules/pathways known to control cell migration are ERK, PI3K-AKT, and p38 [7]. To assess the involvement of these signaling molecules in chemokine-induced migration of the various tumor cells, we tested CXCL12- and CXCL11-dependent chemotactic responses in the presence of the Erk inhibitor, PD98059, the PI-3 K inhibitor, Ly294002, and the p38 inhibitor, SB203580 (Fig. 3). CXCL12- and CXCL11-induced migration of A772 and MDA-MB-231 cells was abolished by LY294002, but not by the other inhibitors. By contrast, CXCL12- and CXCL11-induced migration of A549 and DLD-1 cells was sensitive to both Ly294002 and PD98059. In addition, CXCL11-induced chemotaxis of A549 cells was prevented by SB203580. In case of A767 cells, CXCL12-induced chemotaxis was sensitive to Ly294002, whereas CXCL11-dependent chemotaxis was sensitive to SB203580. Sensitivity of chemotactic responses to the various signaling pathway inhibitors remained unchanged when cells were exposed to a combination of CXCL11 and CXCL12 (additional file 5). These findings establish that CXCL12 and CXCL11 control migration of a given tumor cell either via the same or via distinctly different intracellular signaling molecules/pathways. Our observations further suggest that synergistic migratory effects only occur in cells in which CXCL11- and CXCL12-dependent chemotaxis is mediated via different signaling pathways and in which CXCL11 leads to the activation of p38-signaling.

### Evaluation of combined effects of CXCL11 and CXCL12 on tumor cell invasion

**Fig. 4:**
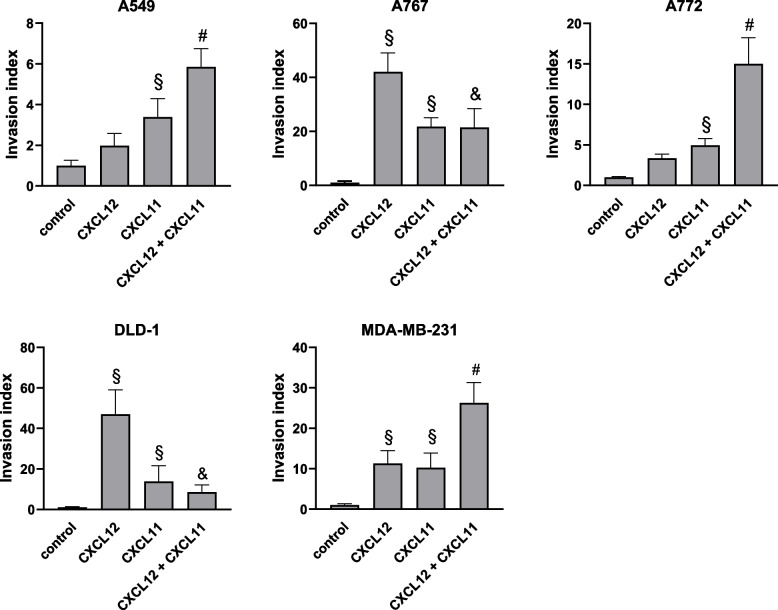
Combined effects of CXCL12 and CXCL11 on tumor cell invasion. CXCL12 (100 ng/ml)- and/or CXCL11 (100 ng/ml)-induced invasion of tumor cells was analyzed using the Boyden chamber assay in combination with membranes coated with ECM proteins (Matrigel). Cell numbers were determined after 72 h. Number of cells invading the membrane in the absence of chemokines was set to 1. Invasion index was calculated as the ratio of cells invading the membrane in the presence and absence of chemokines. Data show average invasion index (+SD) as determined in 5–7 experiments. CXCL12 and CXCL11 promote invasion of tumor cells with different potencies. CXCL12 in combination with CXCL11 additively promote invasion A549, A772, and MDA-MB-231 cells. In A767 and DLD-1 cells, the simultaneous presence of CXCL11 attenuates the stimulatory influences of CXCL12 on cell invasion. § *p* < 0.05 treatment vs. control; # *p* < 0.05 combined treatment vs. CXCL12 and CXCL11 alone; & *p* < 0.05 combined treatment vs. CXCL12

The observed combined effects of CXCL12 and CXCL11 on cell migration prompted us to determine further whether similar applies for cell invasion, a process more closely reflecting metastatic behavior of tumor cells [18]. To this end, we determined CXCL12- and CXCL11-induced cell migration through filters, previously coated with ECM proteins (Fig. 4). CXCL11 (100 ng/ml) promoted invasion of all tumor cells. CXCL12 induced invasion of A767, DLD-1, and MDA-MB-231 cells. Although, CXCL12 also tended to increase the invasion index of A549 and A772 cells, these increases were statistically not significant. Most prominent increases in cell invasion occurred with CXCL12 in A767 and DLD-1 cells, whereas CXCL11 was 50 to 70% less potent in inducing invasion of these cells. Again, FBS (10%) more potently stimulated invasion of all tumor cells (A549, 239 + 19-fold; A767, 508 + 74-fold; A772, 68 + 15-fold; DLD-1, 17 + 3-fold; MDA-MB-231, 6.6 + 1.3-fold; *n* = 3–5). The combined application of CXCL12 and CXCL11 had additive effects on the invasion of A549 cells, and synergistically affected invasion of A772, and MDA-MB-231 cells (Fig. 4). Surprisingly, the effects of a combination of CXCL12 and CXCL11 on invasion of A767 and DLD-1 cells were distinctly smaller than those of CXCL12 alone and rather equaled the effects seen with CXCL11 (Fig. 4). Collectively, these findings establish that CXCL12 and CXCL11 additively or synergistically promote invasion of some tumor cells whereas in other tumor cells CXCL11 seems to suppress the stimulatory effects of CXCL12 on cell invasion.

To further determine whether these diverse cellular responses are reflected by the differential use of CXCR4, CXCR3, and CXCR7, we analyzed CXCL12- and/or CXCL11-induced cell invasion in the presence of the receptor antagonists, AMD3100, AMG487 or CCX771. Invasive responses of all cells to CXCL12 were sensitive to both AMD3100 and CCX771. The invasive response of A767, A772 and DLD-1 cells to CXCL11 was attenuated by AMG487 and CCX771, whereas CXCL11-induced invasion of A549 and MDA-MB-231 cells was only attenuated by AMG487 (additional files 2 and 6). These findings imply that similar to cell migration, differences in the invasive responses of the various cells to chemokines are not determined by chemokine receptors / receptor combinations employed.

### The combined effects of CXCL11 and CXCL12 on tumor cell invasion do not involve gelatinases

To assess whether the combined effects of CXCL12 and CXCL11 on tumor cell invasion involve MMPs as an intermediate, we focused on the gelatinases MMP-2 and MMP-9, which represent known targets of CXCL12 and CXCL11 [19, 20]. Gelatin zymography demonstrated high levels of pro MMP-9 and the absence of pro MMP-2 in MDA-MB-231-derived medium. The supernatant of A767 cells contained low levels of pro MMP-9 and pro MMP-2 while the supernatant of A549 cells only contained pro MMP-9. Both gelatinases were undetectable in medium conditioned by A772 and DLD-1 cells (additional file 7). In all tumor cells, levels of pro MMP-9 and pro MMP-2 remained unchanged following treatment with either CXCL12 or CXCL11 alone (100 ng/ml, 24 h) or in combination. Moreover, activated MMP-9 and activated MMP-2 were absent from all conditioned media. Confirming the findings from zymography, MMP-9 mRNA was detectable in A767 (30 cycles) and MDA-MB-231 (25 cycles) cells by RT-PCR whereas MMP-2 mRNA could only be amplified from A767 (30 cycles) and A549 cells (29 cycles). Moreover, mRNA levels of both MMP-2 and MMP-9 again remained unaffected by chemokines. Together, these findings oppose a crucial role of MMP-9 and MMP-2 in the observed combined effects of CXCL12 and CXCL11 on tumor cell invasion.

### Evaluation of combined effects of CXCL11 and CXCL12 on tumor cell survival

In an attempt to define putative combined effects of CXCL12 and CXCL11 on tumor cell survival, we initially exposed the various cells to either temozolomide (A767, A772), cisplatin (A549, DLD-1) or doxorubicin (MDA-MB-231) at the indicated concentrations and determined numbers of apoptotic cells after 3 h–24 h by immunocytochemical detection of cleaved-caspase-3 (additional files 8 and 9). Based on the results of this analysis, cell cultures were exposed to cisplatin at 20 μM, temozolomide at 100 μM, and doxorubicin at 1 μM for 6-12 h in all following experiments.

**Fig. 5:**
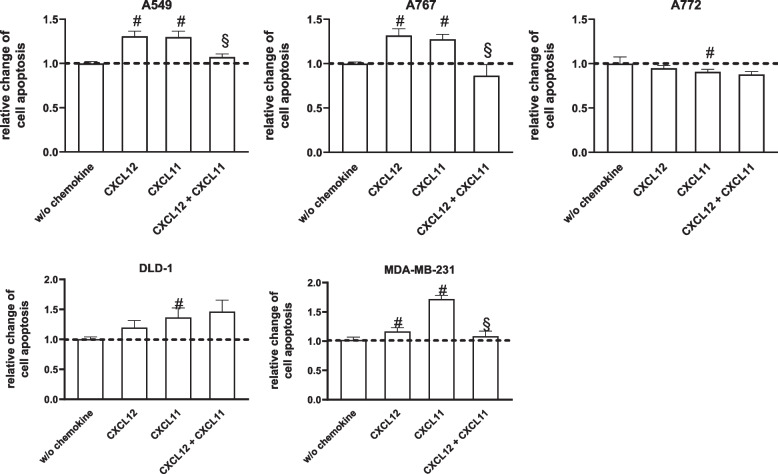
Combined effects of CXCL12 and CXCL11 on toxin-induced tumor cell death. Tumor cells were pretreated with CXCL12 (100 ng/ml), CXCL11 (100 ng/ml), or a combination of both chemokines for 24 h, and subsequently exposed for another 6 h (A767, A772, MDA-MB-231, DLD-1) or 12 h (A549) to either temozolomide (A767, A772; 100 μM), cisplatin (A549, DLD-1; 20 μM) or doxorubicin (MDA-MB-231, 1 μM) in the continuous presence of chemokines. Apoptotic cells were identified by FITC-labeled Annexin V and quantified by flow cytometry. Fluorescence detected in cultures treated with cytostatics in the absence of chemokines was set to 1. Data show relative changes in apoptotic cell death (+ SD) as determined in 3–6 experiments. CXCL12 and CXCL11 attenuated apoptosis of A772 cells, but stimulated death of the other tumor cells. In case of A549, A767, and MDA-MB-231 cells pro-apoptotic effects did not occur following exposure of the cells to a combination of CXCL12 and CXCL11. # *p* < 0.05, presence vs. absence of chemokines; § *p* < 0.05, double treatment vs. single treatment

To assess the effects of chemokines on cytostatic-induced cell death, cell cultures were maintained with either CXCL12 or CXCL11 alone or in combination 24 h prior to the addition of cytostatics as well as during the entire treatment with cytostatic agents (Fig. 5). In 4 out of 5 tumor cell types (A549, A767, DLD-1, MDA-MB-231) CXCL12 and/or CXCL11 induced a slight, but in most cases statistically significant increase in apoptotic cell numbers (Fig. 5). Moreover, in A549, A767, and MDA-MB-231 cells, but not in DLD-1 cells, this increase did not occur following combined treatment with CXCL12 and CXCL11 (Fig. 5). In case of A772 cells, CXCL12, CXCL11, and the combination of both chemokines slightly attenuated apoptotic cell death. To exclude that analysis is biased by chemokine-induced cell proliferation, we maintained the various tumor cells with CXCL12, CXCL11 or both for 48 h and determined numbers of proliferating cells by BrdU-labeling. None of the treatments promoted cell proliferation (additional file 10). Staining with 7-AAD further showed that chemokines do not affect numbers of cells residing within different phases of the cell cycle (additional file 10).

We conclude that CXCL12 and CXCL11 commonly have pro-apoptotic effects on tumor cells, which in most cases are absent in the simultaneous presence of the chemokines. We further conclude that CXCL12, CXCL11 alone or in combination exert anti-apoptotic effects only in distinct tumor cells.

## Discussion

The CXCL12- and CXCL11-systems are intimately interweaved as CXCL12 and CXCL11 signal through CXCR4/CXCR7 and CXCR3/CXCR7, respectively [7]. Our previous work revealed that CXCL12 and CXCL11 promote tumor cell migration with equal potency and further demonstrated that in most tumor cells these migratory responses involve either CXCR7 alone or CXCR7 in combination with another chemokine receptor [6, 15]. Since many tumors show increased expression of both CXCL12 and CXCL11 [14], we now asked whether CXCL12 and CXCL11 would exert combined effects on tumor progression. We demonstrate the existence of complex interplay between the CXCL11- and CXCL12-chemokine systems, which differs with respect to tumor cell type and cellular function.

Despite the established interaction between the CXCL12- and CXCL11-systems, combined effects of both chemokine systems on tumor growth and metastasis are still ill defined. The few available studies so far demonstrated that a combination of CXCL12 and CXCL11 either potentiated or diminished tumor cell migration when compared to single chemokines [21, 22]. Along this line, our present more complex analysis of different human tumor cells and cellular responses revealed that depending on the cell type, combined influences of CXCL12 and CXCL11 range from enhanced effects on cell migration and invasion to attenuated cell invasion and cell apoptosis or the complete absence of combined influences.

Intriguingly, we were unable to correlate the distinct mode of interaction on tumor cell migration with the previously established use of distinct CXCL12 and CXCL11 receptors in the migratory response of the respective tumor cells [6, 15] (additional files 2 and 4). For example, whereas in both A549 and A772 cells, CXCL12 and CXCL11 promote cell migration through CXCR7 and CXCR3/CXCR7, respectively, a combination of (medium concentrations) of CXCL12 and CXCL11 showed additive effects only on the migration of A549 cells, but not of A772 cells (Fig. 1). Such a correlation could also not be established for cell invasion. For example, CXCL11 and CXCL12 synergistically promote invasion of A772 and MDA-MB-231 cells. In both cells, CXCL12 induces invasion via CXCR4 and CXCR7. However, CXCL11-dependent invasion requires CXCR3 and CXCR7 in A772 cells, but only CXCR3 in MDA-MB-231 cells (additional files 2 and 6).

Interestingly, synergistic effects of CXCL12 and CXCL11 on cell migration were only detectable in tumor cells in which CXCL11-dependent cell migration was sensitive to the p38 inhibitor, SB203580 (A549, A767), and in which CXCL11 and CXCL12 affected cell migration via different signaling pathways. Whether this implies that synergistic migratory responses require the concomitant activation of p38-signaling and either Erk- or PKA-signaling needs further analysis. Again, chemokine-induced p38-signaling did not correlate with the use of distinct chemokine receptors in the migratory responses of the respective cells (see additional files 2 and 4). We further wish to note that although in none of our tumor cells CXCL12-induced cell migration was sensitive to SB203580 this does not imply that the CXCL12-p38-axis is not involved in the control of cell migration. In fact, previous work highlighted the importance of p38 signaling in the migratory response of both normal and malignant cells to CXCL12 [23–27].

Synergistic effects on cell migration were only detectable with CXCL11 and CXCL12 at concentrations (1 ng/ml – 10 ng/ml, equals about 0.12–1.2 nM) distinctly lower than usually applied to induce maximal cellular responses in vitro (100 ng/ml). The physiological relevance of these findings is favored by the known serum concentrations of CXCL11 and CXCL12 ranging between 0.1 ng/ml − 1 ng/ml [28]. Moreover, by using an in vivo model a more recent work demonstrated optimal chemotactic response along gradients from 0 to 12 nM of CXCL12 [29].

Human CXCR3 exists in the splice variants, CXCR3A and CXCR3B as well as the truncated variant, CXCR3alt. Whereas CXCR3A exerts pro-tumorigenic influences by increasing cell migration/invasion and proliferation/survival, CXCR3B rather allows for anti-tumorigenic effects, including reduced cell migration/invasion and proliferation/survival [30]. The CXCR3alt-variant only results in very low activation of cell signaling and, hence, is often considered as signaling-deficient [31]. Several lines of evidence currently oppose the possibility that the observed diverse migratory, invasive, and apoptotic responses of the various tumor cells to a combination of CXCL12 and CXCL11 would be due to the differential use of CXCR3A and CXCR3B. Although our previous work revealed that A549, DLD-1 and MDA-MB-231 cells express CXCR3B, we obtained no indication for the involvement of CXCR3B in the migratory responses of these tumor cells to CXCL11 [6]. Likewise, in none of our present experiments CXCL11 and CXCL12 had opposing effects on a given cell function, which would be indicative for the involvement of different CXCR3 splice variants. Finally, it is noteworthy that CXCL11 binds CXCR3B with about 100 times lower affinity than CXCR3A [32] and, hence, preferentially activates CXCR3A.

In contrast to cell migration, cell invasion requires the additional cleavage of basal membrane and extracellular matrix proteins; this fact most likely reflects the requirement of different receptors / receptor combination in the control of cell migration versus cell invasion. Protein cleavage is commonly achieved by MMPs [33], especially the gelatinases, MMP-2 and MMP-9 [34]. MMP-2 and/or MMP-9 are targets of CXCL12 and CXCL11, which promote their expression [19, 20]. Whereas MMP-9 is highly upregulated in most types of cancer, MMP-2 expression only modestly increases [35]. Quantitative RT-PCR and gelatin zymography demonstrated low to medium expression levels of MMP-2 and/or MMP-9 in some tumor cells and their absence in others. Moreover, in none of the tumor cells, gelatinase expression was affected by CXCL12 and/or CXCL11, implying that gelatinases do not account for co-operative effects of these chemokines on tumor cell invasion. This conclusion, however, disregards the potential role of other MMPs involved in tumor spreading [36].

Chemokines, including CXCL12 and CXCL11, are commonly assumed to have anti-apoptotic activity and, hence, are thought to prevent chemically induced cell death [37–40]. So far, the only known exceptions are acute myeloid leukemia cells, which show increased apoptosis following treatment with CXCL12 [41, 42]. Interestingly, the majority of tumor cells tested (4 out of 5) showed increased toxin-induced cell death in the presence of either CXCL12 or CXCL11. Only in one type of tumor cells (A772), chemokines slightly attenuated cell death. This implies that pro-apoptotic effects of chemokines are more widespread than previously assumed. Moreover, in most, but not all cases, enhanced cell death was undetectable and, thus, pro-apoptotic activities of CXCL12 and CXCL11 were neutralized when both chemokines were present. Unfortunately, the chemokine-dependent signaling pathways/events leading to tumor cell survival or cell death are currently not well characterized, hence, preventing any hint to the molecular mechanism(s) underlying the diverse survival responses. Notably, the diverse survival responses were unrelated to chemokine-induced cell proliferation, which was undetectable in cells treated with either CXCL12 or CXCL11 alone or in combination.

While our present studies focused on direct effects of CXCL12 and CXCL11 on tumor cell functions, it is important to keep in mind that CXCL12 and CXCL11 additionally affect tumor progression by controlling immune cell invasion and function as well as tumor angiogenesis via modulating the tumor microenvironment [43, 44]. Data on combined effects of CXCL12 and CXCL11 on the tumor microenvironment are at present unavailable. The existence of such combined influences is nevertheless likely and might eventually render further complexity to the crosstalk of CXCL12 and CXCL11 in tumor progression.

## Conclusions

Our present demonstration that depending on the tumor cell type, a combination of CXCL12 and CXCL11 either allows for pro- or anti-tumor activity put caution on ongoing efforts to establish chemokine receptors as therapeutic targets in cancer [45]. These findings oppose the standardized inactivation of (distinct) chemokine receptors in cancer patients and rather argue in favor of personalized targeting approaches.

## Supplementary Information

**Additional file 1.**List of primary and secondary antibodies.

**Additional file 2.** Overview of chemokine receptors mediating chemotactic and invasive responses of tumor cells to CXCL11 and CXCL12.

**Additional file 3.a** Chemokine receptor expression by glioma cells. **b** Over-exposed full blots from which bands shown in additional file 3a have been cropped at shorter exposure time. Blots were not cut prior to incubation with antibodies.

**Additional file 4.** Chemokine receptors mediating chemotactic influences of CXCL12 and CXCL11 in A767 and A772 cells.

**Additional file 5.** Effects of signaling pathway inhibitors on chemotactic responses of tumor cells to a combination of CXCL11 and CXCL12.

**Additional file 6.** Identification of chemokine receptors mediating invasive responses of tumor cells to CXCL11 and CXCL12.

**Additional file 7.a** Chemokines fail to affect gelatinase expression in tumor cells. **b** Full gels of zymography assay. For description, see Additional file 7a.

**Additional file 8.** Time course of cytostatic-induced death of cancer cells.

**Additional file 9.** Dose-dependency of cytostatic-induced death of cancer cells.

**Additional file 10.** CXCL11 and/or CXCL12 remain without effects on tumor cell proliferation.

## Data Availability

The datasets used and/or analyzed during the current study are available from the corresponding author on reasonable request.

## Abbreviations

ANOVA: One way analysis of variance
FBS: Fetal bovine serum
MMP: Matrix metalloproteinase
PBS: Phosphate-buffered saline
qRT-PCR: quantitative real-time-PCR
