# Correction: Interactions of the chemokines CXCL11 and CXCL12 in human tumor cells

**DOI:** 10.1186/s12885-023-11276-5

**Published:** 2023-08-18

**Authors:** Christian Koch, Nina Charlotte Fischer, Malte Puchert, Jürgen Engele

**Affiliations:** 1 Institute of Anatomy, Medical Faculty, University of Leipzig, Liebigstr. 13, 04103 Leipzig, Germany; 2 Department of Medical Oncology and Hematology, University of Zurich and University Hospital of Zurich, Raemistrasse 100, Zurich, 8091 Switzerland

**Correction: BMC Cancer 22, 1335 (2022)**

10.1186/s12885-022-10451-4.

Following publication of the original article [1], the authors identified that additional file 5 was repeated as both additional file 4 and 5. The correct additional file 4 is supplied in this correction article.

### Electronic supplementary material

Below is the link to the electronic supplementary material.

**Additional file 4.** Chemokine receptors mediating chemotactic influences of CXCL12 and CXCL11 in A767 and A772 cells.
