# Supplementary material for: Interactions of the chemokines CXCL11 and CXCL12 in human tumor cells
Source: BMC Cancer. 2022 Dec 20;22:1335. doi: 10.1186/s12885-022-10451-4 (PMC9768901; doi:10.1186/s12885-022-10451-4)
Supplement: Supplementary file 1 — Additional file 1.List of primary and secondary antibodies. [file 12885_2022_10451_MOESM1_ESM.pdf]

Additional file 1

List of primary and secondary antibodies.

Primary antibodies	Supplier	Species raised in; clonality	Dilution (application)	Reference
CXCR4	Abcam, Cambridge, UK	Goat, polyclonal	1:1000 (WB)	ab1670
CXCR7	Acris, Herford, Germany	Rabbit, polyclonal	1:1000 (WB)	AP17961PU-N
CXCR3	R&D Systems, Minneapolis, MN	Mouse, monoclonal	1:1000 (WB)	clone #49801
cleaved caspase-3 (N175)	Cell Signaling Technologies, Danvers, MA	Rabbit, polyclonal	1:400 (ICC)	#9661
GAPDH	Fitzgerald Industries International, Acton, MA	Mouse, monoclonal	1:5000 (WB)	10RG109A
**Secondary antibodies**	**Supplier**	**Species raised in; clonality**	**Dilution (application)**	**Reference**
Alexa Fluor 488 anti-rabbit	Thermo Fisher Scientific, Waltham, MA	Donkey, polyclonal	1:200 (ICC)	A21206
Alexa Fluor 555 anti-rabbit	Thermo Fisher Scientific	Donkey, polyclonal	1:200 (ICC)	A31572
Peroxidase-conjugated IgG anti-rabbit	Jackson Immunoresearch, West Grove, PA	Donkey, polyclonal	1:10,000 (WB)	711-035-152
Peroxidase-conjugated IgG anti-mouse	Vector Laboratories, Burlingame, CA	Horse	1:10,000 (WB)	PI-2000

WB, Western blot; ICC, immunocytochemistry
