# Supplementary material for: Interactions of the chemokines CXCL11 and CXCL12 in human tumor cells
Source: BMC Cancer. 2022 Dec 20;22:1335. doi: 10.1186/s12885-022-10451-4 (PMC9768901; doi:10.1186/s12885-022-10451-4)
Supplement: Supplementary file 2 — Additional file 2. Overview of chemokine receptors mediating chemotactic and invasive responses of tumor cells to CXCL11 and CXCL12. [file 12885_2022_10451_MOESM2_ESM.pdf]

Additional file 2

Overview of chemokine receptors mediating chemotactic and invasive responses of tumor cells to CXCL11 and CXCL12.

	**A549**	**A767**	**A772**	**DLD-1**	**MDA-231**
Chemotactic response to CXCL11	CXCR3 + CXCR7	CXCR3	CXCR3 + CXCR7	CXCR3	CXCR3 + CXCR7
Chemotactic response to CXCL12	CXCR7	CXCR4 + CXCR7	CXCR7	CXCR7	CXCR4 + CXCR7
Invasive response to CXCL11	CXCR3	CXCR3 + CXCR7	CXCR3 + CXCR7	CXCR3 + CXCR7	CXCR3
Invasive response to CXCL12	CXCR4 + CXCR7	CXCR4 + CXCR7	CXCR4 + CXCR7	CXCR4 + CXCR7	CXCR4 + CXCR7

Data compiled from Puchert et al. (2018; 2020) and the present study.
