# Supplementary material for: Interactions of the chemokines CXCL11 and CXCL12 in human tumor cells
Source: BMC Cancer. 2022 Dec 20;22:1335. doi: 10.1186/s12885-022-10451-4 (PMC9768901; doi:10.1186/s12885-022-10451-4)
Supplement: Supplementary file 3 — Additional file 3.a Chemokine receptor expression by glioma cells. b Over-exposed full blots from which bands shown in additional file 3a have been cropped at shorter exposure time. Blots were not cut prior to incubation with antibodies. [file 12885_2022_10451_MOESM3_ESM.pdf]

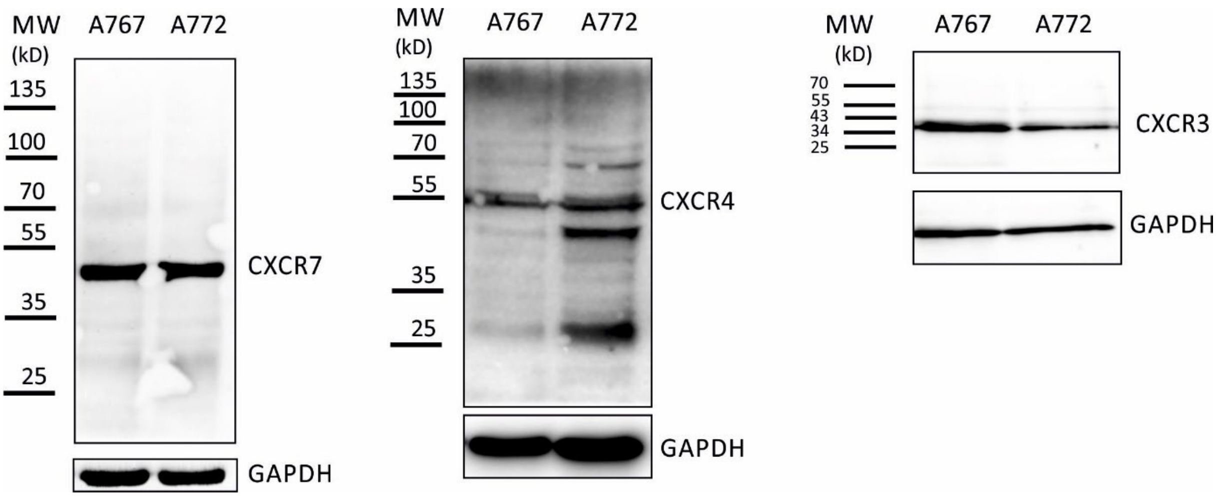

Additional file 3a

Chemokine receptor expression by glioma cells.

Western blot analysis demonstrating that A767 and A772 cells express CXCR7, CXCR4, and CXCR3 to detectable levels. GAPDH served as a loading control. In case of A772, the nature of additional protein bands stained by CXCR4 antibodies are unknown.

CXCR7

MW A549 DLD1 MDA A767 A772

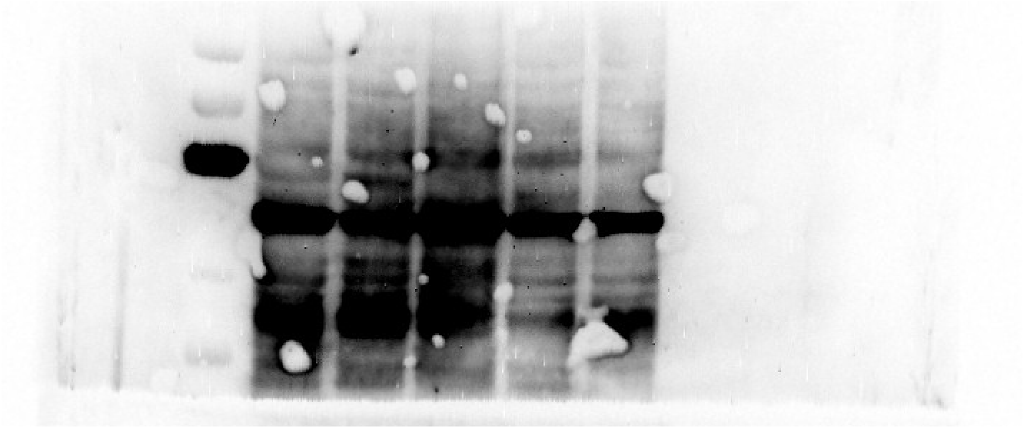

CXCR7 - GAPDH

A549 DLD1 MDA A767 A772

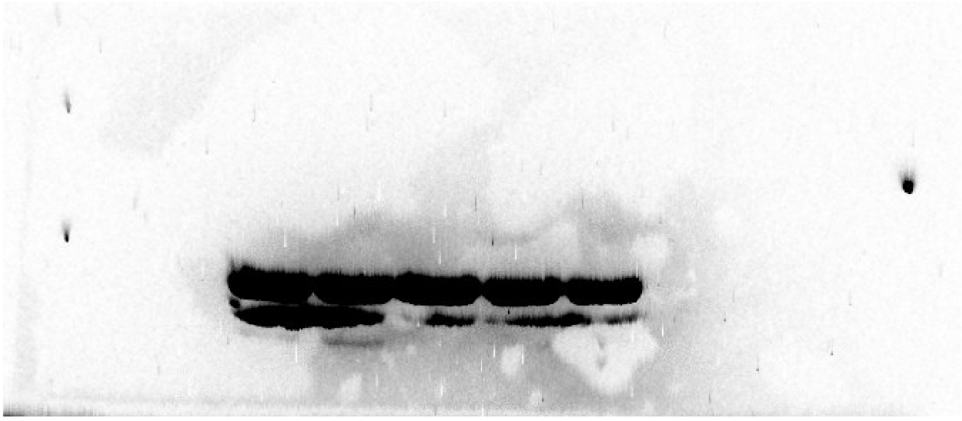

CXCR4

MW A549 DLD1 MDA A767 A772

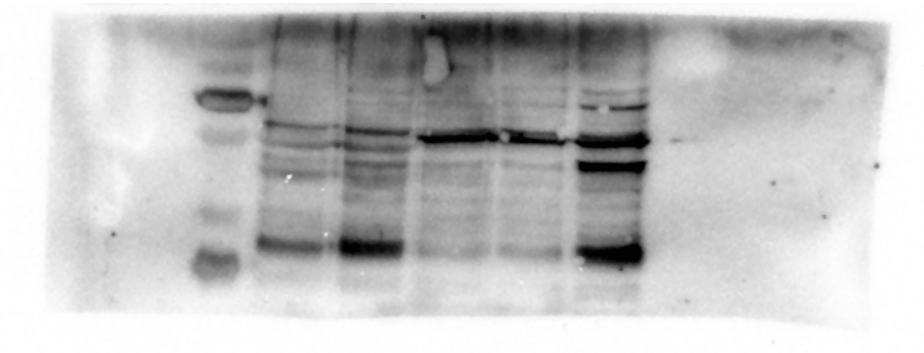

CXCR4 – GAPDH

A549 DLD1 MDA A767 A772

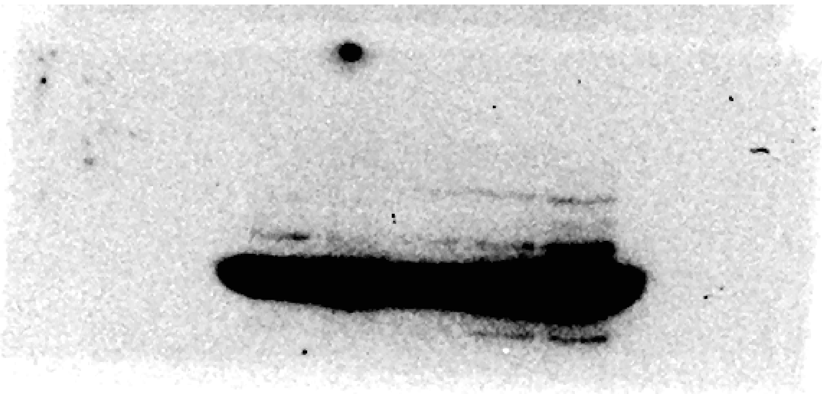

CXCR3

MW A549 DLD1 MDA A 767 A772

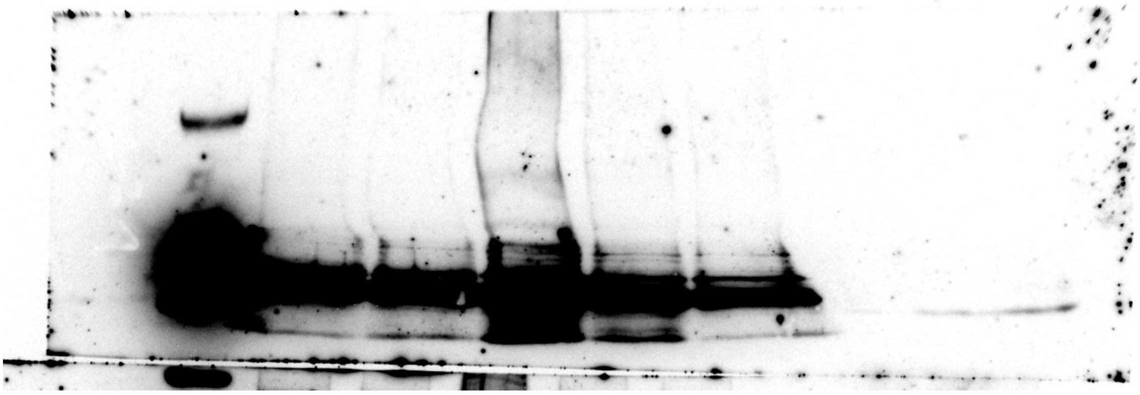

CXCR3 – GAPDH loading control

MW A549 DLD1 MDA A 767 A772

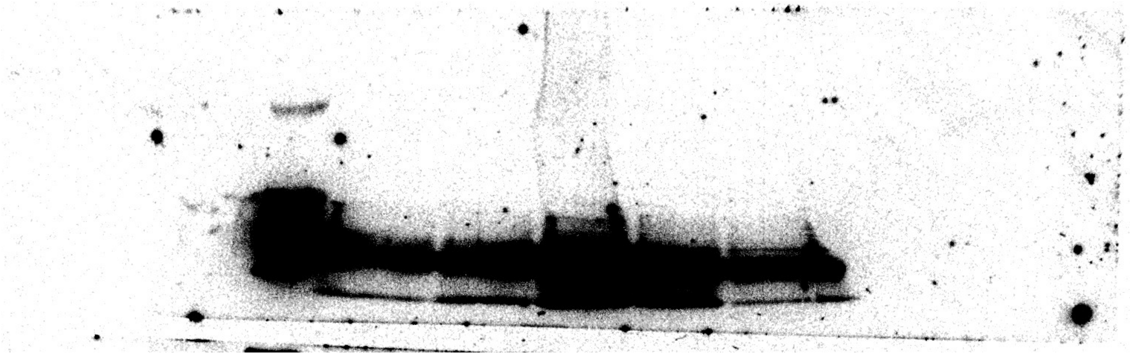

Additional file 3b

Over-exposed full blots from which bands shown in additional file 3a have been cropped at shorter exposure time. Blots were not cut prior to incubation with antibodies.
