# Supplementary material for: Interactions of the chemokines CXCL11 and CXCL12 in human tumor cells
Source: BMC Cancer. 2022 Dec 20;22:1335. doi: 10.1186/s12885-022-10451-4 (PMC9768901; doi:10.1186/s12885-022-10451-4)
Supplement: Supplementary file 5 — Additional file 5. Effects of signaling pathway inhibitors on chemotactic responses of tumor cells to a combination of CXCL11 and CXCL12. [file 12885_2022_10451_MOESM5_ESM.pdf]

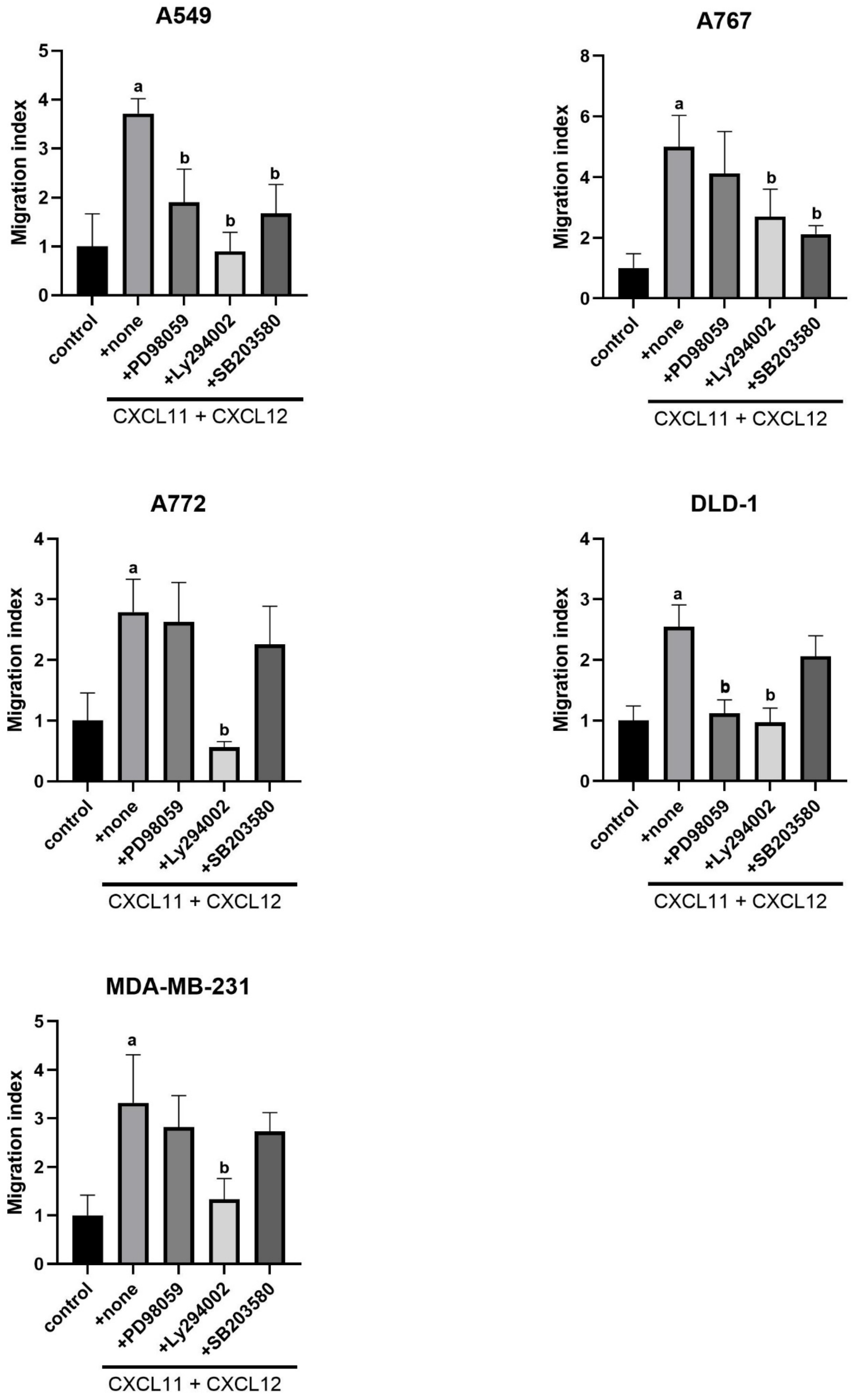
A549
A767
A772
DLD-1
MDA-MB-231
Migration index
a
b
control
+none
+PD98059
+Ly294002
+SB203580
CXCL11 + CXCL12

Additional file 5

Effects of signaling pathway inhibitors on chemotactic responses of tumor cells to a combination of CXCL11 and CXCL12.

Cells were treated with PD98059 (20 µM), LY294002 (20 µM), or SB203580 (10 µM) for 1 h, and subsequently tested for their migratory responses to a combination of CXCL12 (100 ng/ml) and CXCL11 (100 ng/ml) in a modified Boyden chamber. Data represent average migration index (±SD) as determined in 3-9 experiments. Note that signaling pathways mediating chemotactic responses are unaltered in cells exposed to a combination to CXCL11 and CXCL12 when compared to single chemokine (see Fig. 3). [a]$p<0.05$, presence vs. absence of chemokines; [b]$p<0.05$, presence vs. absence of inhibitors.
