# Supplementary material for: Interactions of the chemokines CXCL11 and CXCL12 in human tumor cells
Source: BMC Cancer. 2022 Dec 20;22:1335. doi: 10.1186/s12885-022-10451-4 (PMC9768901; doi:10.1186/s12885-022-10451-4)
Supplement: Supplementary file 6 — Additional file 6. Identification of chemokine receptors mediating invasive responses of tumor cells to CXCL11 and CXCL12. [file 12885_2022_10451_MOESM6_ESM.pdf]

Cells	Chemokine	Inhibitor	% Inhibition +/- SD	n	p-value
A549	CXCL12	AMD3100	73.9 +/- 11.3	8	<0.001
		CCX771	63.9 +/- 22.2	6	<0.001
	CXCL11	AMG481	84.6 +/- 4.8	6	<0.001
		CCX771	32.1 +/- 15.1	6	=0.46
	CXCL12 + CXCL11	AMD3100	42.7 +/- 14.0	5	<0.001
		CCX771	44.4 +/- 17.8	5	<0.001
		AMG487	80.4 +/- 11.4	5	<0.001
A767	CXCL12	AMD3100	78.1 +/- 0.3	3	<0.001
		CCX771	89.1 +/- 2.5	3	<0.001
	CXCL11	AMG487	75.9 +/- 3.1	3	<0.001
		CCX771	80.4 +/- 4.9	3	<0.001
	CXCL12 + CXCL11	AMD3100	72.3 +/- 9.0	4	<0.001
		CCX771	89.4 +/- 3.9	3	<0.001
		AMG487	73.8 +/- 3.0	4	<0.001
A722	CXCL12	AMD3100	92.8 +/- 5.1	5	<0.001
		CCX771	97.1 +/- 3.0	5	<0.001
	CXCL11	AMG481	87.1 +/- 13.1	5	<0.001
		CCX771	98.5 +/- 3.1	6	<0.001
	CXCL12 + CXCL11	AMD3100	94.0 +/- 8.3	6	<0.001
		CCX771	98.3 +/- 4.2	6	<0.001
		AMG487	94.8 +/- 10.1	5	<0.001
DLD-1	CXCL12	AMD3100	91.8 +/- 7.1	6	<0.001
		CCX771	45.3 +/- 5.8	3	<0.001
	CXCL11	AMG481	81.1 +/- 11.2	6	<0.001
		CCX771	62.6 +/- 17.4	6	<0.001
	CXCL12 + CXCL11	AMD3100	85.3 +/- 4.5	5	<0.001
		CCX771	61.3 +/- 8.4	4	<0.01
		AMG487	29.7 +/- 0.2	3	=0.59
MDA-MB-231	CXCL12	AMD3100	93.0 +/- 7.3	5	<0.001
		CCX771	90.5 +/- 10.2	7	<0.001
	CXCL11	AMG481	46.1 +/- 11.1	6	<0.01
		CCX771	-0.57 +/- 26.6	5	>0.99
	CXCL12 + CXCL11	AMD3100	85.2 +/- 7.3	7	<0.001
		CCX771	64.2 +/- 14.7	6	<0.001
		AMG487	61.5 +/- 16.7	6	<0.001

Additional file 6

Identification of chemokine receptors mediating invasive responses of tumor cells to CXCL11 and CXCL12.

Cells were treated for 1 h with AMD3100 (10 µM); CCX771 (100 nM), or AMG481 (10 µM) and subsequently analyzed for CXCL12 (100 ng/ml)- and/or CXCL11 (100 ng/ml)-induced cell invasion using a Boyden chamber in which membranes were coated with Matrigel. Cell numbers were determined after 72h. Effects of receptor antagonists are expressed as % inhibition (mean ± SD) of cell invasion occurring in their absence. Note the cell type-specific use of chemokine receptors for mediating invasive responses to CXCL11 and CXCL12.
