# Supplementary material for: Interactions of the chemokines CXCL11 and CXCL12 in human tumor cells
Source: BMC Cancer. 2022 Dec 20;22:1335. doi: 10.1186/s12885-022-10451-4 (PMC9768901; doi:10.1186/s12885-022-10451-4)
Supplement: Supplementary file 7 — Additional file 7.a Chemokines fail to affect gelatinase expression in tumor cells. b Full gels of zymography assay. For description, see Additional file 7a. [file 12885_2022_10451_MOESM7_ESM.pdf]

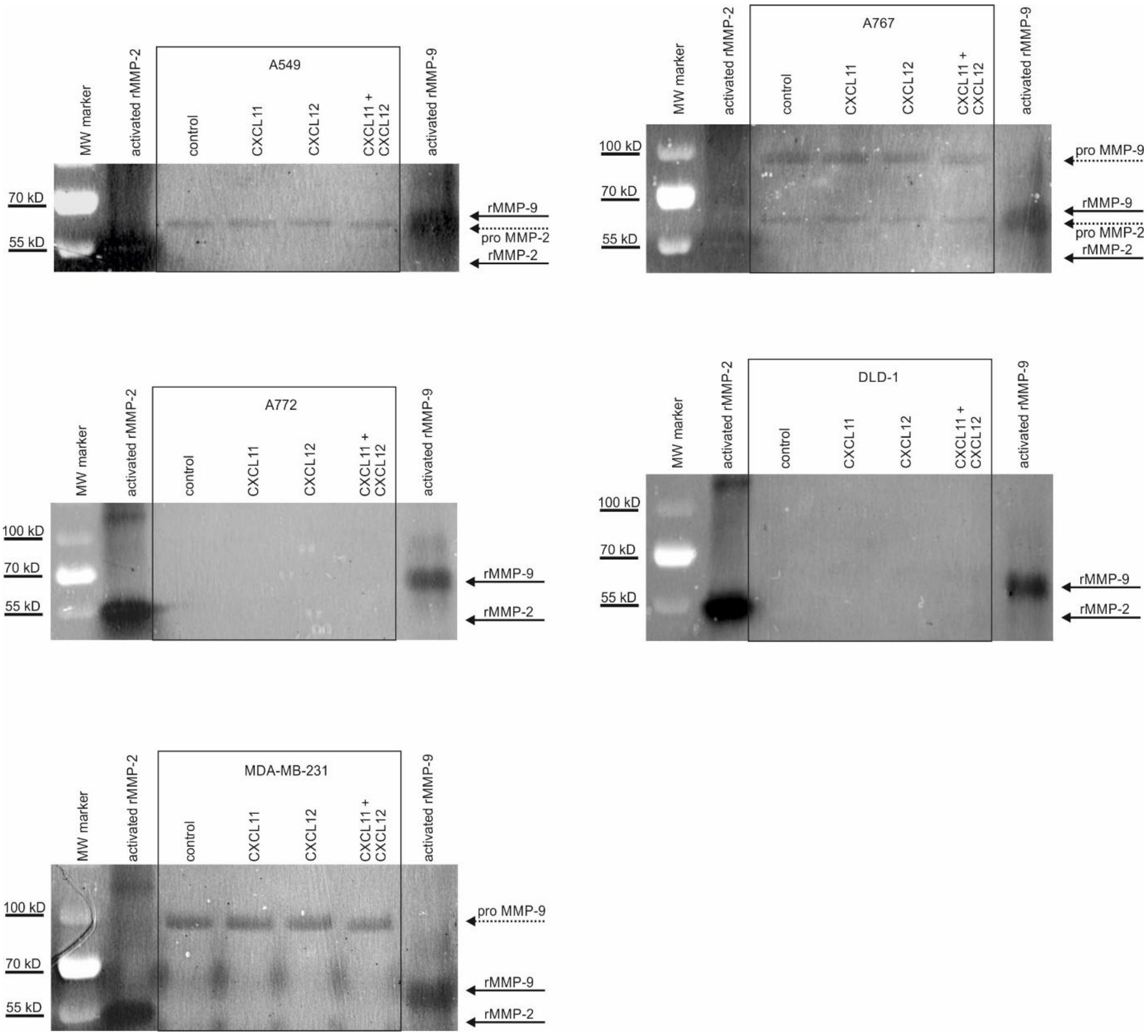

Additional file 7a

Chemokines fail to affect gelatinase expression in tumor cells.

A549, A767, A772, DLD-1, and MDA-MB-231 cells were maintained with serum-free culture medium and treated with CXCL11 (100 ng/ml), CXCL12 (100 ng/ml) or both for 48h. The cell supernatant was collected and analyzed for MMP-2 and MMP-9 expression by gelatin zymography. To this end, the concentrated supernatant was separated on a gelatin-containing polyacrylamide gel under non-denaturating conditions and exposed to enzymatic activity for 24h. Following staining with Coomassieblue, gelatin-free areas appear as pale bands, which have been electronically inversed. Activated recombinant human MMP-2 and MMP-9 served as controls. Expression of pro MMP-9 and/or pro MMP-2 was detectable in A549, A767, and MDA-MB-231 cells and was not affected by chemokines. None of the cells showed detectable levels of activated gelatinases.

A549

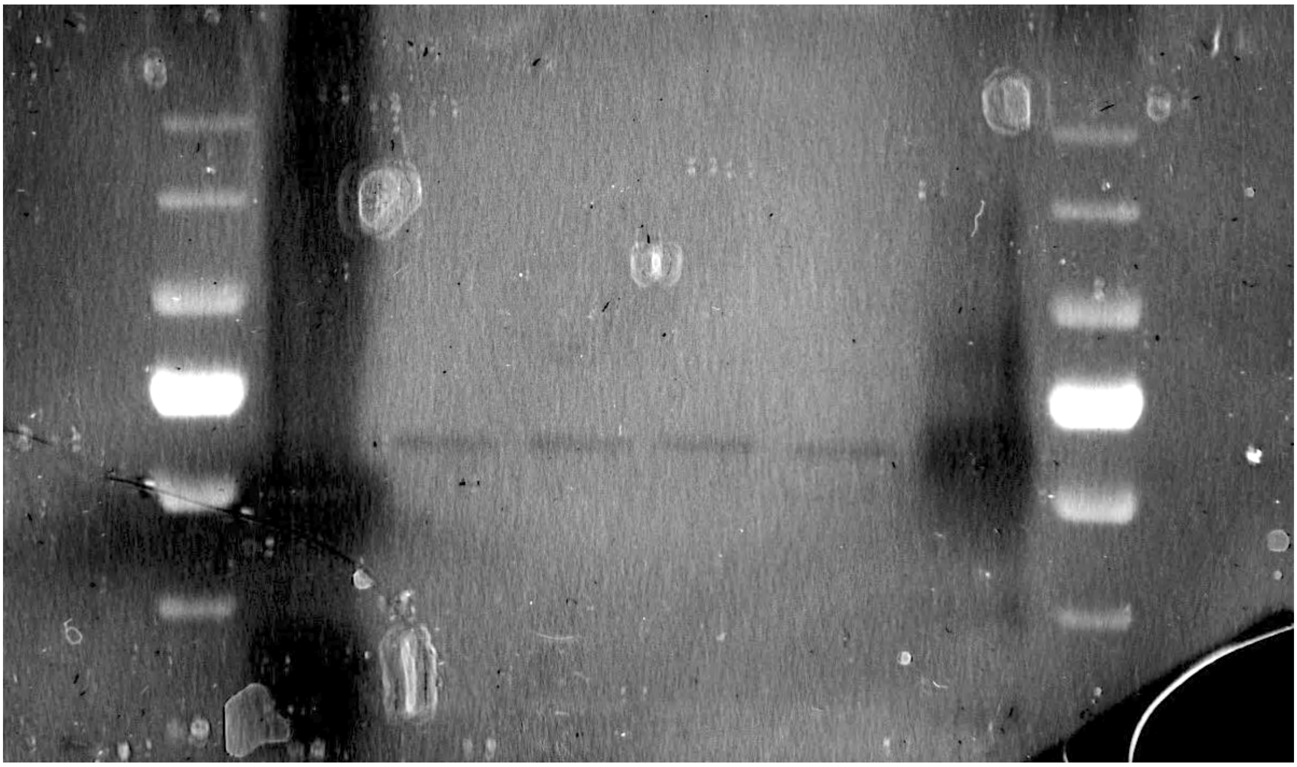

A767

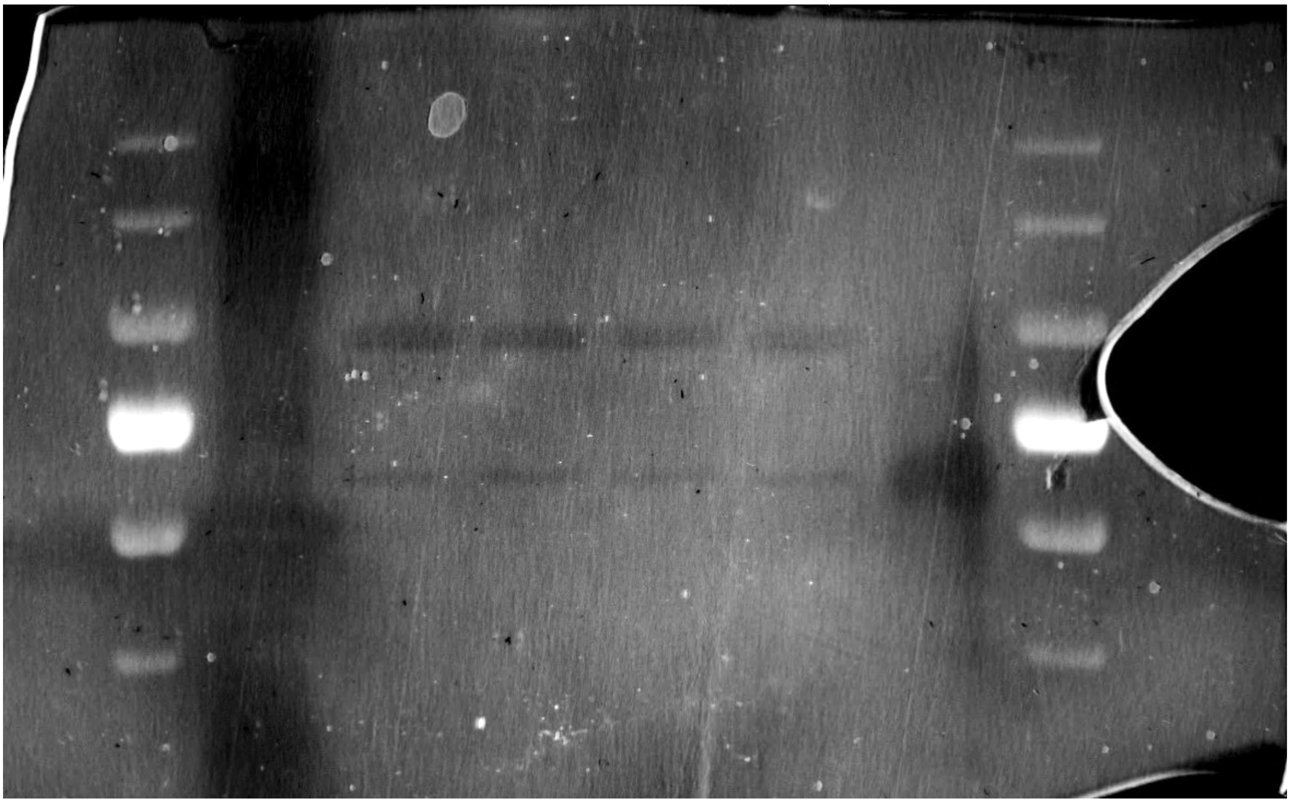

A772

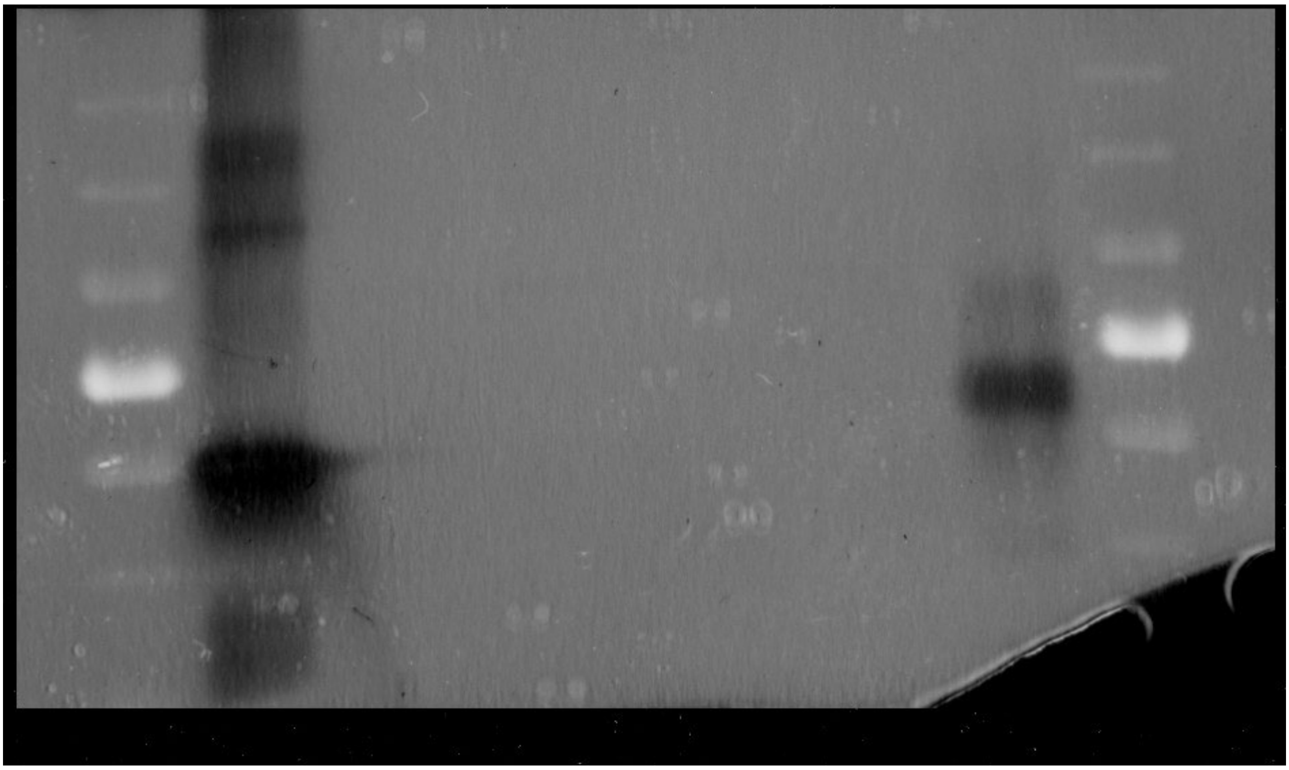

DLD-1

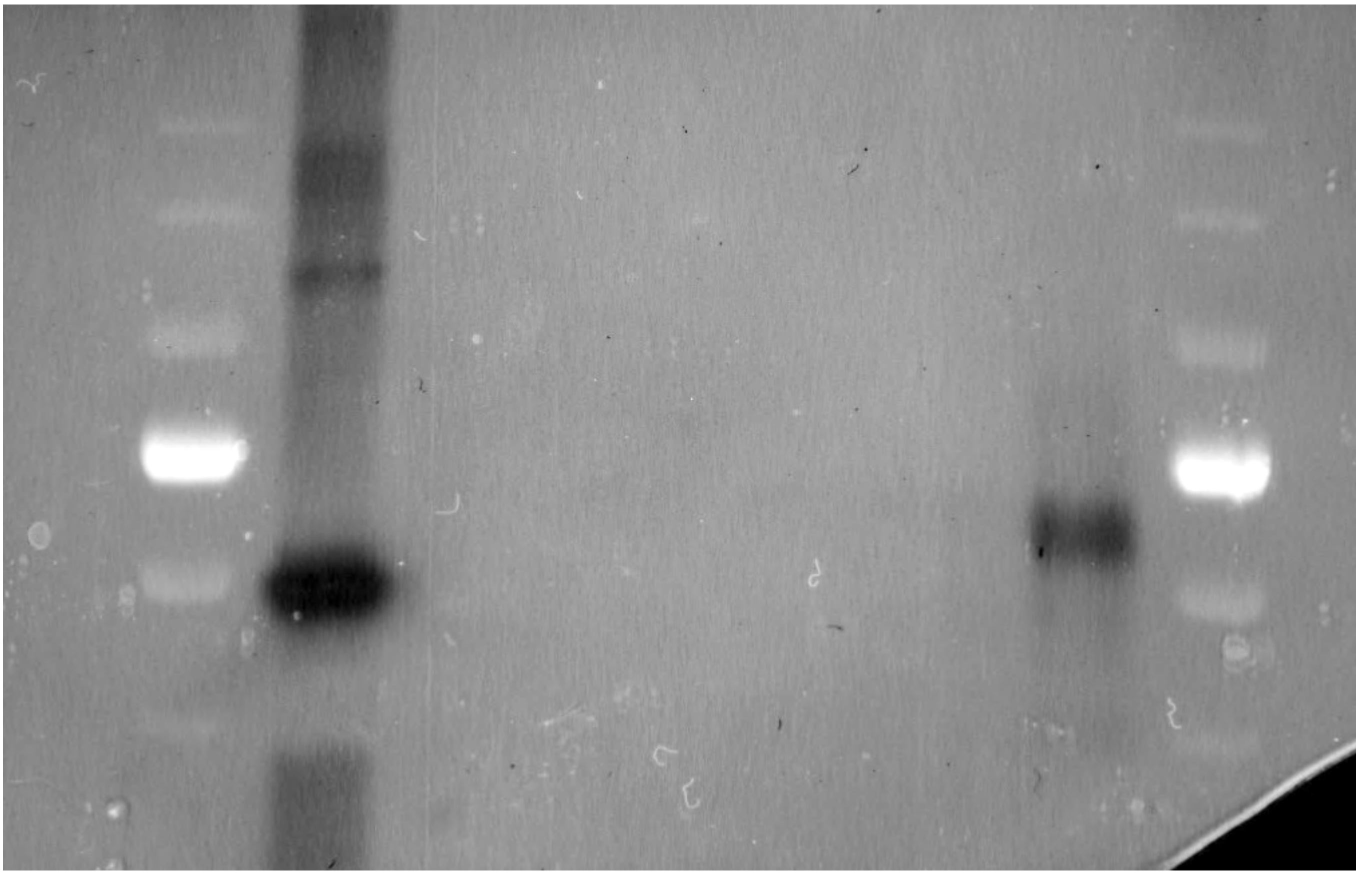

MDA-MB-231

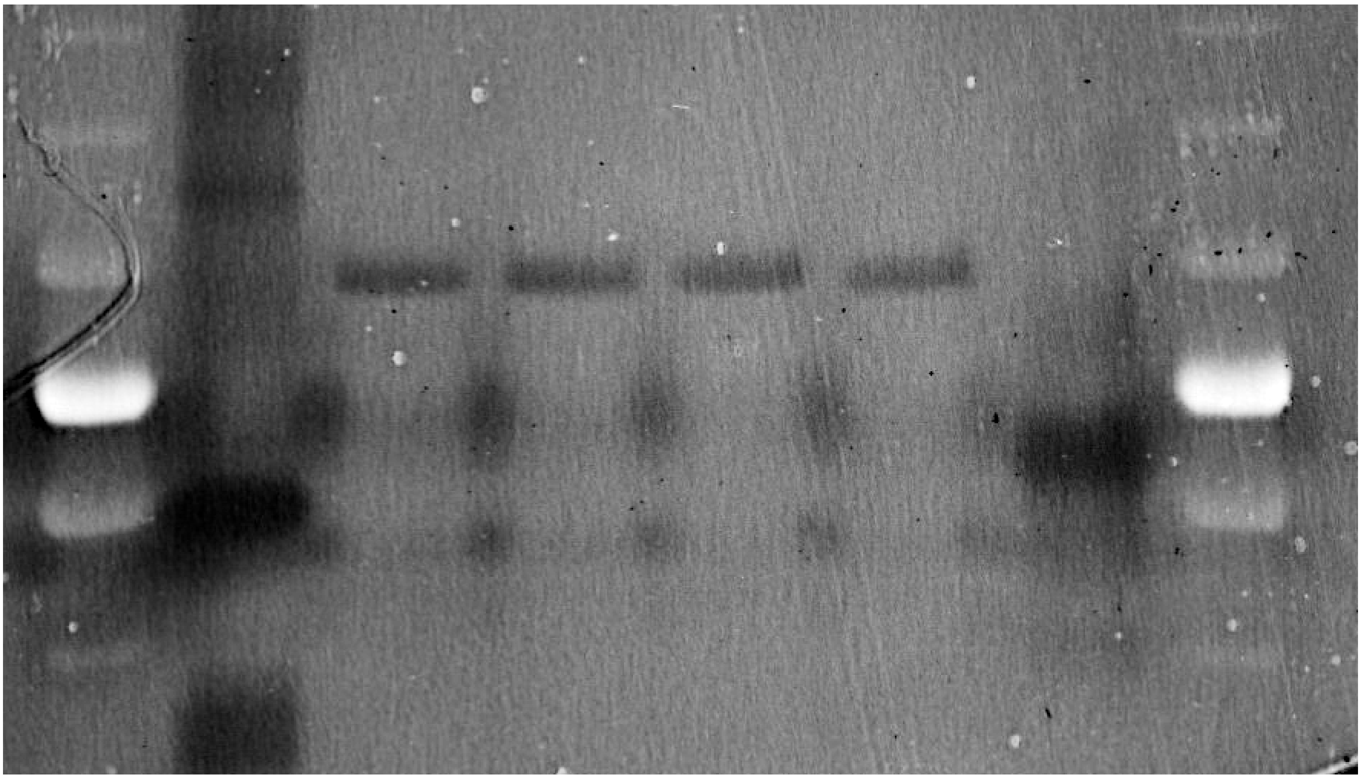

Additional file 7b

Full gels of zymography assay. For description, see additional file 7a.
