# Supplementary material for: Interactions of the chemokines CXCL11 and CXCL12 in human tumor cells
Source: BMC Cancer. 2022 Dec 20;22:1335. doi: 10.1186/s12885-022-10451-4 (PMC9768901; doi:10.1186/s12885-022-10451-4)
Supplement: Supplementary file 8 — Additional file 8. Time course of cytostatic-induced death of cancer cells. [file 12885_2022_10451_MOESM8_ESM.pdf]

Additional file 8

Time course of cytostatic-induced death of cancer cells.

	**3h**	**6h**	**12h**	**24h**
**A549 + cisplatin (20 µM)**				
mean (%)	0	1	18	97
SD	0	1	9	5
n	3	5	3	4
**A767 + temozolomide (100µM)**				
mean (%)	29	28	22	17
SD	4	11	17	7
n	4	4	4	3
**A772 + temozolomide (100 µM)**				
mean (%)	45	45	45	32
SD	4	14	7	4
n	5	5	4	3
**DLD-1 + cisplatin (20 µM)**				
mean (%)	0	21	28	100
SD	0	6	5	2
n	3	5	5	3
**MDA-MB-231 + doxorubicin (1 µM)**				
mean (%)	23	47	99	100
SD	5	8	1	1
n	5	5	3	3

Tumor cells were treated with cytostatics for 3h to 24h as indicated. Cells were subsequently fixed and stained with antibodies against cleaved caspase-3. Immunopositive (apoptotic) and immunonegative cells were counted in at least 3 observation fields on a Zeiss confocal laser scan microscope and expressed as the ratio (%) of dead to living cells.
