# Supplementary material for: Interactions of the chemokines CXCL11 and CXCL12 in human tumor cells
Source: BMC Cancer. 2022 Dec 20;22:1335. doi: 10.1186/s12885-022-10451-4 (PMC9768901; doi:10.1186/s12885-022-10451-4)
Supplement: Supplementary file 9 — Additional file 9. Dose-dependency of cytostatic-induced death of cancer cells. [file 12885_2022_10451_MOESM9_ESM.pdf]

Additional file 9

Dose-dependency of cytostatic-induced death of cancer cells

	**Concentration**		
**A549 + cisplatin (12h)**	**10 µM**	**20 µM**	**40 µM**
mean (%)	12	18	31
SD (%)	2	10	2
n	4	3	5
**A767 + temozolomide (6h)**	**50 µM**	**100 µM**	**200 µM**
mean (%)	22	28	56
SD (%)	9	12	13
n	5	4	4
**A772 + temozolomide (6h)**	**50 µM**	**100 µM**	**200 µM**
mean (%)	48	45	56
SD (%)	9	14	11
n	6	5	5
**DLD-1 + cisplatin (6h)**	**10 µM**	**20 µM**	**40 µM**
mean (%)	10	21	14
SD (%)	4	6	4
n	4	5	5
**MDA-MB-231 + doxorubicin (6h)**	**0.1 µM**	**1 µM**	**10 µM**
mean (%)	11	47	100
SD (%)	6	8	2
n	6	5	4

Tumor cells were treated with cytostatics at the indicated concentrations and the ratio (%) of caspase-3-immunoreactive (apoptotic) cells was determined after 6-12h as described in additional file 8.
