# Supplementary material for: Interactions of the chemokines CXCL11 and CXCL12 in human tumor cells
Source: BMC Cancer. 2022 Dec 20;22:1335. doi: 10.1186/s12885-022-10451-4 (PMC9768901; doi:10.1186/s12885-022-10451-4)
Supplement: Supplementary file 10 — Additional file 10. CXCL11 and/or CXCL12 remain without effects on tumor cell proliferation. [file 12885_2022_10451_MOESM10_ESM.pdf]

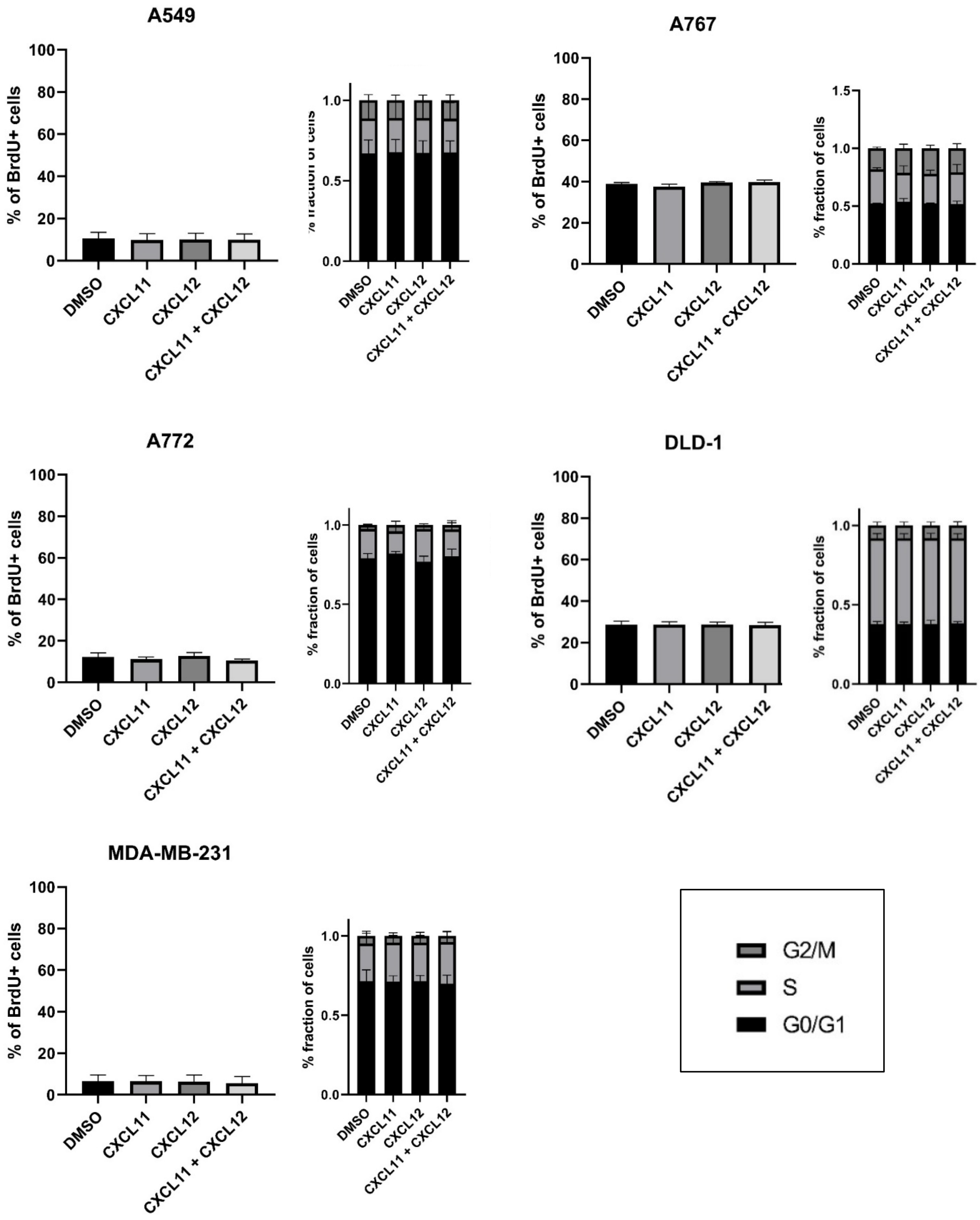
A549
A767
A772
DLD-1
MDA-MB-231
% of BrdU+ cells
% fraction of cells
DMSO
CXCL11
CXCL12
CXCL11 + CXCL12
G2/M
S
G0/G1

Additional file 10

CXCL11 and/or CXCL12 remain without effects on tumor cell proliferation.

Cells were maintained with serum-free medium supplemented with CXCL11 (100ng/ml) and/or CXCL12 (100ng/ml) for 24h. The final 2h, medium was additionally supplemented with bromodeoxyuridine (10 µM). For FACS analysis, cells were stained for BrdU and DNA was labelled with 7-AAD. Date show average fraction (%) ±SD of labeled cells as determined in 4-6 experiments.
