# Supplementary material for: Correction: Interactions of the chemokines CXCL11 and CXCL12 in human tumor cells
Source: BMC Cancer. 2023 Aug 18;23:769. doi: 10.1186/s12885-023-11276-5 (PMC10436660; doi:10.1186/s12885-023-11276-5)
Supplement: Supplementary file 1 — Additional file 4. Chemokine receptors mediating chemotactic influences of CXCL12 and CXCL11 in A767 and A772 cells. [file 12885_2023_11276_MOESM1_ESM.pdf]

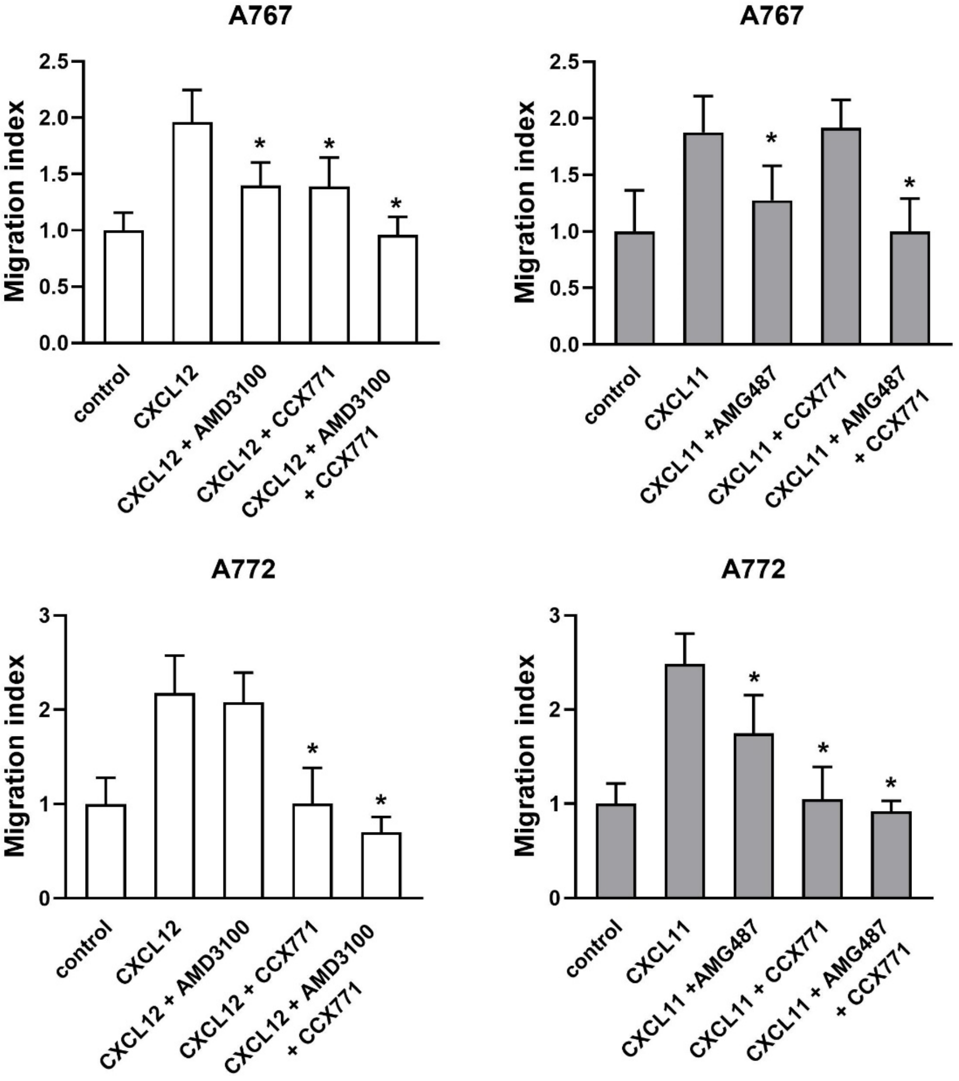

Additional file 4

Chemokine receptors mediating chemotactic influences of CXCL12 and CXCL11 in A767 and A772 cells.

A767 and A772 cells were incubated with the CXCR3 antagonist, AMG487 (10 µM), the CXCR4 antagonist, AMD3100 (10 µM) and/or the CXCR7 antagonist, CCX771 (100 nM) for 1h and subsequently tested for CXCL12 (100 ng/ml)- or CXCL11 (100 ng/ml)-induced cell migration in a modified Boyden chamber. Number of cells migrating in the absence of chemokines was set to 1. Migration index was calculated as the ratio of cells migrating in the presence and absence of chemokines. Bars show means $\pm$ SD (n = 6-10). CXCL12-dependent migration of A776 cells requires both CXCR4 and CXCR7, whereas CXCL12-dependent migration solely depends on CXCR7. CXCL11-induced migration of A767 and A772 cells is mediated by CXCR3 and CXCR3/CXCR7, respectively.*p<0.05, presence vs. absence of antagonists.
